# VPS35-Based Approach: A Potential Innovative Treatment in Parkinson's Disease

**DOI:** 10.3389/fneur.2019.01272

**Published:** 2019-12-17

**Authors:** Simona Eleuteri, Alberto Albanese

**Affiliations:** ^1^Department of Neurology, Humanitas Research Hospital, Milan, Italy; ^2^Department of Neurology, Catholic University, Milan, Italy

**Keywords:** endosomal trafficking, retromer complex, therapeutic targets, Parkinson's disease, alpha-synucein, amyloid protein A (AA)

## Abstract

Several symptomatic treatments for Parkinson's disease (PD) are currently available. Still, the challenge today is to find a therapy that might reduce degeneration and slow down disease progression. The identification of pathogenic mutations in familial Parkinsonism (fPD) associated to the monogenic forms of PD provided pathophysiological insights and highlighted novel targets for therapeutic intervention. Mutations in the VPS35 gene have been associated with autosomal dominant fPD and a clinical phenotype indistinguishable from idiopathic PD. Although VPS35 mutations are relatively rare causes of PD, their study may help understanding specific cellular and molecular alterations that lead to accumulation α-synuclein in neurons of PD patients. Interacting with such mechanisms may provide innovative therapeutic approaches. We review here the evidence on the physiological role of VPS35 as a key intracellular trafficking protein controlling relevant neuronal functions. We further analyze VPS35 activity on α-synuclein degradation pathways that control the equilibrium between α-synuclein clearance and accumulation. Finally, we highlight the strategies for increasing VPS35 levels as a potential tool to treat PD.

## Introduction

Parkinson's disease (PD) is the second most common neurodegenerative disorder affecting 1% of people over 65 and 4.3% of those older than 85 (1).

The clinical hallmark of PD is bradykinesia, in combination with rest tremor and rigidity (1). This phase is preceded by a preclinical stage, when neurodegenerative processes have commenced, but there are no evident symptoms or signs, and by a prodromal phase, when symptoms and signs are present, but are yet insufficient to define disease (2). The motor phenomenology is attributed to the selective loss of dopaminergic neurons (DNs) in the substantia nigra pars compacta (SNpc) resulting in degeneration of the nigrostriatal tract and dopamine deficiency (3).

**Figure 1:**
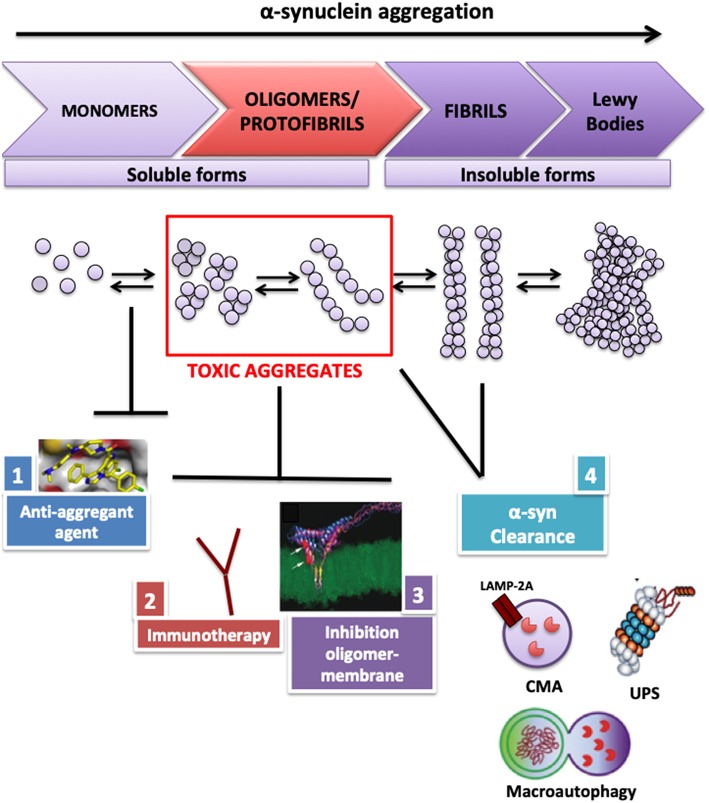
Therapeutic strategies used to reduce α-synuclein toxic aggregates accumulation in neurons. To reduce α-synuclein accumulation in neurons, several therapeutic approaches have been evaluated and tested in murine PD models: (1) antiamyloidogenic agents, such as small molecules or biocomposts to block α-synuclein aggregation and fibrillogenesis; (2) immunotherapy using α-synuclein single-chain antibody able to attenuate neuronal degeneration *in vivo*; (3) peptide design to block the interaction between α-synuclein oligomer and membrane, able to improve deficits in murine PD models; (4) boosting α-synuclein degradation pathways. CMA, chaperone mediated autophagy; UPS, ubiquitin proteosome system; LAMP2A, lysosome-associated membrane protein 2A.

The neuropathological hallmark of PD is the occurrence of intracellular inclusions, Lewy bodies (LBs) and Lewy neurites, mainly consisting of aggregated α-synuclein (4). A remarkably convincing set of data supports the view that accumulation and aggregation of α-synuclein in neurons may represent a key pathogenic event (5–9). Several approaches aim to reduce the accumulation and propagation of α-synuclein toxic aggregates in the brain (10–12) (Figure 1). Ideally, an intervention aimed to reduce α-synuclein should be performed in the preclinical disease phase or—at least—in the prodromal phase, although it may be argued that the early clinical phase may also provide a suitable time window.

Monogenic PD subtypes represent a powerful tool for understanding the pathogenesis of PD and for developing treatment strategies. Mutations of the VPS35-retromer subunit have been recently shown to cause PD and to play a key role in neurodegenerative processes, as they control balance between degradation and recycling of fundamental proteins for neuronal survival (13). Moreover, this review outlines the genetic and biochemical evidence that pathogenic missense mutation and depletion of VPS35 affect dopamine (DA) neurons function at different levels. More recently, a direct link has been established between VPS35 and α-synuclein in a rodent PD model where VPS35 deficiency causes α-synuclein accumulation (14). This effect of VPS35 could unravel its key role in controlling macroautophagy, chaperone-mediated autophagy (CMA), and lysosomal function, which are the main degradation pathways of α-synuclein (15–17). A growing body of data converges to establish how VPS35 controls the transport and the localization of protein markers involved in α-synuclein degradation pathways, thus linking VPS35 to α-synuclein accumulation (18–21). An increase in VPS35 levels in PD mice rescues α-synuclein accumulation and induces neuroprotection (14), pointing to a possible role of VPS35 as a therapeutic target for PD. Moreover, VPS35 could represent a diseases-modifying target, and its neuroprotective role could be due to the control on neuronal signaling events (22), synaptic plasticity (23–25), trafficking of protein in dendritic spines (26), membrane dopamine transporter (DAT) recycling in DA neurons (27), and the regulation mitochondrial functions (28).

## Pathogenic Role Of VPS35 In PD

**Figure 2:**
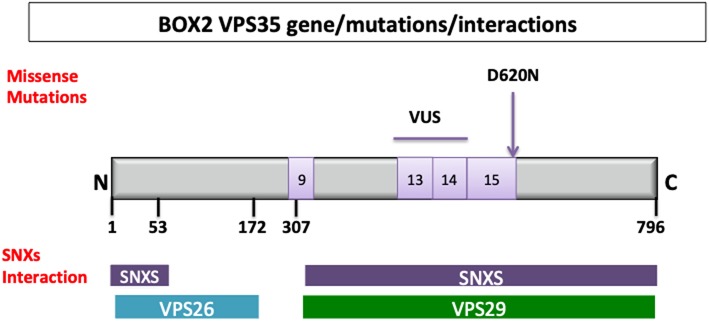
VPS35 gene, protein structure and interactions. Retromer cargo recognition complex is formed by vacuolar sorting protein 26 (VPS26; 38 kDa), vacuolar sorting protein 29 (VPS29; 20 kDa), vacuolar sorting protein 35 (VPS35; 92 kDa); the dimer of sinexin (SNXs). The N-terminal region (in blue) from the amino acid residues 1 to 172 [1–172] and C-terminal region (violet) from 307 to 796 (307–796) are important, respectively, for the interaction with VPS26 and VPS29 (36). The amino acid residues involved in the interaction with the SNXs are at N-terminal region (1–35, 37–54) and C-terminal region (307–796). A structural level VPS35 is a right-handed α-helix solenoid, and it is predicted to have 34 helices, 13 of which are in C-terminal. Different missense mutations have been identified in fPD. VPS35 variant of uncertain significance (VUS) are localized between exon 9 (P316S), exon 13 (R542W), and exon 14 (I560T, H599R, M607V), and the confirmed pathogenic mutation (D620N) is localized on the exon 15 (genes in light violet).

Two independent studies with exome sequencing identified point mutations in the VPS35 gene causing an autosomal dominant form of PD (PARK17) (29, 30). The p.Asp620Asn (c.1858G>A) mutation has been proven to be pathogenic with a frequency ~1.3% in familial cases and 0.3% in sporadic PD cases (31, 32). In addition, non-pathogenic VPS35 variants (33) and variant of uncertain significance (34) have been described. The VPS35 gene maps on chromosome 16q11.2 encompasses 29.6 kb and 17 exons (35) (Figure 2).

The clinical presentation of PARK17 PD patients bearing pathogenic VPS35 gene mutations is that of a typical clinically definite PD. The mean age at disease onset is 50.3 ± 7.3 years; cognitive and psychiatric features do not predominate, and levodopa response is sustained and not associated to severe dyskinesias (37).

To evaluate the pathogenic the role of VPS35 deletion and the VPS35–D620N mutation, different transgenic mouse models have been developed. Knockout mice with the deletion of the endogenous VPS35 at embryonic stage resulted in early lethality under embryonic day 10 (38). VPS35 heterozygote mice (expressing 50% of protein respect to the wild type) survived to adulthood (38) and did not presented any PD-relevant deficit up to 6 months of age. However, the neuropathological evaluation at late stage (12 months) in VPS35 heterozygote mice revealed DA neuronal loss in SNpc and increase in α-syn levels (16). VPS35^DAT−CRE^ mice demonstrated as the depletion of VPS35 protein in SNpc-DA neurons at 2–3 months of age already showed a reduction in TH^+^ DA neurons and TH^+^ fibers in the striatum (28). To evaluate the effect of VPS35–D620N mutation, a viral-mediated gene transfer model in adult rat has been developed, showing that the expression of the mutant in the nigrostriatal pathway was sufficient to induce DA neuronal loss and axonal pathology (39). VPS35–D620N knockin (KI) mice (40) at 3 months of age have shown normal motor functions and no loss of striatal DA neurons (41, 42). However, it has been reported that KI mice showed increased capacity to evoke dopamine release in dorsolateral striatum, consistent with elevated extracellular dopamine (42). The evaluation of the effect of VPS35–D620 KI mice on PD pathology with chronic aging provided evidence that the increase in the endogenous D620N mutant levels has a gain of function or partial-negative dominant mechanism (43). Thus, the endogenous expression of VPS35–D620N is sufficient to recapitulate some features of PD pathology inducing (i) the progressive degeneration of the nigrostriatal pathway, (ii) modest motor deficit with age consistence, and (iii) widespread axonal damage (43). In VPS35–D620N KI mice, α-synuclein accumulation and LB pathology were not detected, but tau-positive pathology, characterized by the abnormal accumulation of early “pretangle,” has been described (43). Moreover, VPS35–D620N KI mice or knockdown or knockout of VPS35 mice impacted LRRK2-mediated Rab protein phosphorylation (44) showing that VPS35 could play a major role in controlling LRRKK2 kinase activity and supporting the hypothesis that VPS35–D620N mutation resulted in a gain of function (44).

## VPS35 Subunit Of the Retromer Complex

The VPS35 gene encodes for a subunit of the retromer complex that is composed of 796 amino acids with a molecular weight of 92 kDa (45). The retromer is an evolutionary conserved complex of eukaryotic cells that structurally comprises two subcomplexes: (i) a cargo recognition complex VPS26–VPS29–VPS35 heterotrimer mainly involved in the transport of proteins and (ii) a membrane-targeting dimer of sorting nexin, important for the adhesion of the complex to the endosomal membrane (SNX1, SNX2, SNX5, SNX6, and SNX32) (45) (Figure 2). Mammals have two paralogues of VPS26 subunit (VPS26A and VPS26B) that compete for a single binding site on VPS35 (46). Two variants of VPS26A have been identified in a patient with familial PD (46).

**Figure 3:**
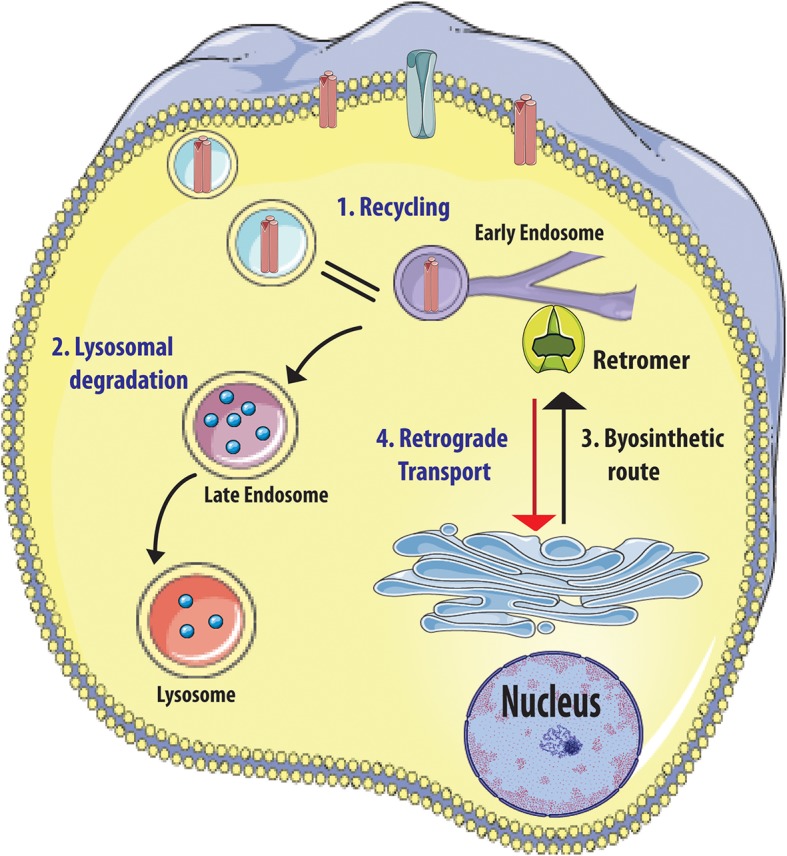
Retromer complex functions. Retromer complex (in green) controls the sorting of cargoes (1) originate from plasma membrane (receptors in red), (2) direct to lysosome for degradation, and (3) trafficking between trans-Golgi Network (TGN) (3, 4). Figure was adapted from Servier Medical Art images (http://smart.servier.com/).

The retromer complex represents the master “conductor” of the sorting in the endosomal network; it is localized at the early endosome (EE) and mainly controls the retrograde trafficking of cargo proteins from EE to the trans-Golgi network (TGN) or to the plasma membrane (Figure 3). Moreover, the retromer complex mediates vesicular transport from mitochondria to peroxisomes (47). This complex represents the main sorting hub that receives, dissociates, and sorts cargoes of different origin: (i) plasma membrane (recycling of membrane receptors), (ii) biosynthetic pathways (retrieval of trafficking from Golgi), and (iii) lysosomal pathway (cargoes direct to lysosomes) (48) (Figure 3). Overall, these activities control the homeostasis of transmembrane proteins at plasma membrane and endolysosomal levels and regulate receptor abundance, signaling receptors, adhesion molecules, and hydrolase receptors.

## The Retromer Complex Controls Synaptic Function

It has been recently clarified that the retromer complex plays a key role in neurons, where it can, directly or indirectly, mediate essential neuronal functions through cargo selector accessory proteins (SNX27 and WASH complex). Thus, the retromer influences neuronal signaling events (such as downregulation of receptors activity) (22), synaptic plasticity (23–25), trafficking of protein in dendritic spines (26), and membrane DAT recycling in DA neurons (27). The VPS26-retromer subunit belonging to the β-arrestin family controls the downregulation and recycling to cell surface of β_2_-adrenergic receptor A (22). This is a G-protein-coupled receptor characterized by a cyclical pattern of activation/inactivation through endosomal internalization: the retromer balances the recycling and the degradation of this receptor (49). The retromer could also influence neuronal activity indirectly, controlling function and localization of accessory proteins, such as SNX27 and WASH complex. The SNX27 protein controls recycling of the α-amino-3-hydroxy-5-methyl-4-isoxazolepropionic acid receptor, involved in long-term potentiation, a process linked to learning and memory (23, 25). The D620N VPS35 pathogenic mutation impairs interaction with the WASH complex (15) and may lead to mistrafficking protein cargoes, such as the GluA1 glutamate receptors subunit, the Glut-1 glucose transporter (26, 49, 50) and in α-amino-3-hydroxy-5-methyl-4-isoxazolepropionic acid receptors (51). DAT, which is strictly associated to the activity of DA neurons, undergoes constitutive internalization: its recycling is mediated by the retromer complex (27).

Therefore, retromer disruption via short hairpin RNA-mediated VPS35 knockdown results in DAT depletion from plasma membrane and decrease in DAT recycling (27). In summary, the retromer influences trafficking of signaling receptors and controls rapid signaling events involved in synaptic plasticity and dendritic spine formation that are specifically relevant to DA neuron function (27).

## VPS35 Influences α-Synuclein Accumulation

Recent studies highlighted the crucial role of the retromer complex in controlling the accumulation of α-synuclein in PD mouse models and in an *in vitro* model of α-synuclein spreading (14). Reduction in VPS35 levels or the expression of mutated VPS35 protein in PD mouse hippocampus displayed defective α-synuclein clearance resulting in widespread accumulation of aggregates (14). These data were confirmed in mouse models showing that VPS35 deficiency or a pathogenic VPS35 mutation leads to accumulation and aggregation of α-synuclein in the SN, accompanied by degeneration of DA neurons, reduction in DA levels, impairment of locomotor behavior, and alteration of lysosomal morphology (16). On the contrary, an excess of wild-type VPS35 expression rescues the accumulation of α-synuclein aggregates and leads to the reduction in neuronal loss and astrogliosis in a PD mouse model overexpressing α-synuclein (14). Using an *in vitro* model of cortical neurons, it has been demonstrated that an increase in wild-type VPS35 levels may counteract the propagation of α-synuclein aggregates from neuron to neuron (14). In keeping with this line of evidence, VPS35 ablation in *Drosophila* results in the accumulation of α-synuclein in lysosomes (17). Furthermore, silencing of endogenous VPS35 in an α-synuclein transgenic fly does not only promote the accumulation of human wild-type α-synuclein but also causes eye degeneration and motor disability (17). These data suggest that endosomal dysfunction caused by VPS35 deficiency impairs the ability of neurons to cope with the accumulation of α-synuclein, thus facilitating the spread of PD pathology.

## How Does VPS35 Control α-Synuclein Accumulation?

The hypothesis that VPS35 could promote α-synuclein clearance is related to the observation of a central role of this retromer protein in controlling interaction and transport of key proteins in α-synuclein degradation pathways.

**Figure 4:**
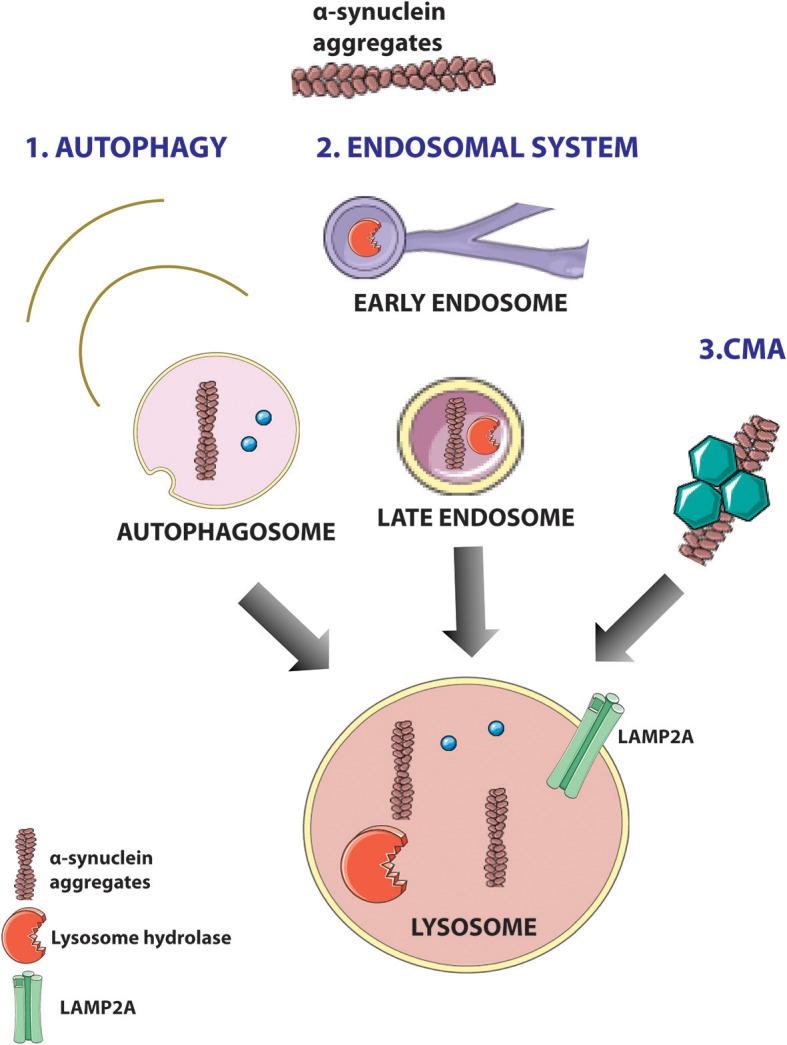
α-Synuclein degradation pathways in cells. Toxic aggregates of α-synuclein can be degraded in the cells through three pathways: macroautophagy (1), endosomal system (2), and chaperone-mediated autophagy (CMA) (3). (1) In the autophagy, key molecules can lead the autophagosome formation and the degradation of aggregates of α-synuclein; (2) lysosomal hydrolases generated in trans-Golgi Network (TGN) are sorted from specific hydrolase sorting receptors [cation-independent 6 mannose phosphate receptor (CI-MPR) and Sortilin], through the endosomal system to the lysosome, and mediate α-synuclein degradation; (3) chaperone-dependent selection of soluble α-synuclein aggregates that are targeted to lysosomes and directly translocated across the lysosomal membrane for degradation. Figure was adapted from Servier Medical Art images (http://smart.servier.com/).

**Figure 5:**
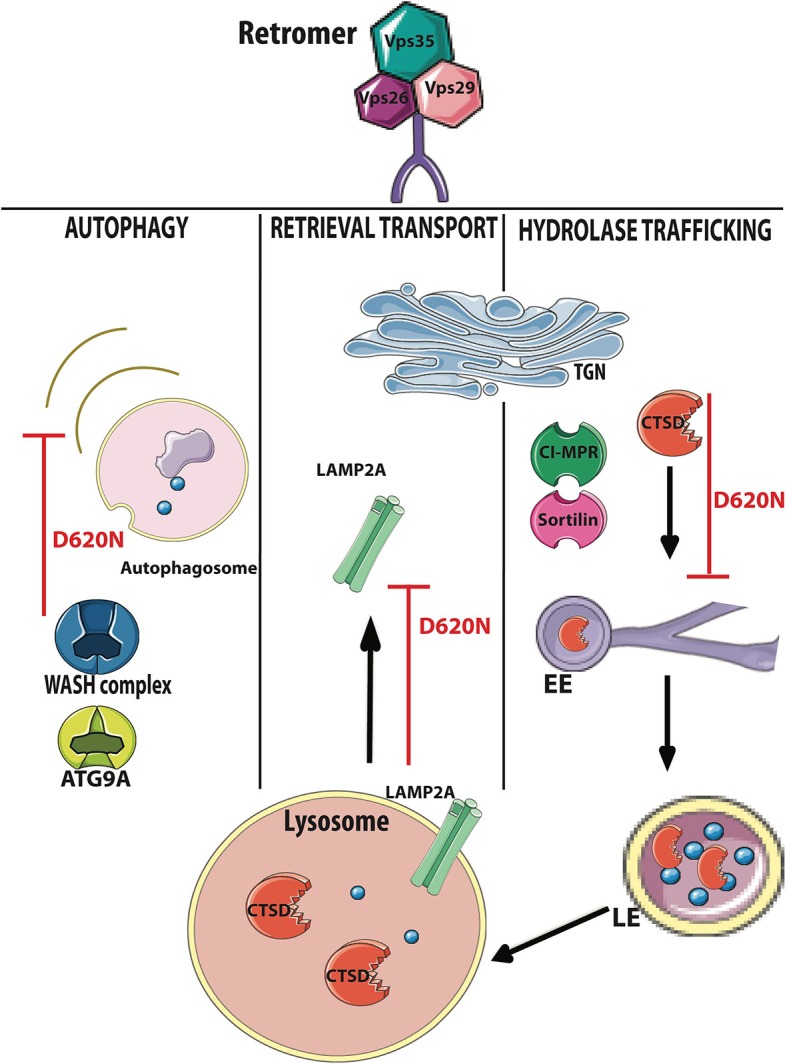
VPS35-retromer subunit controls the main α-synuclein degradation pathways. VPS35 has a relevant role in controlling the degradation of key molecules in the α-synuclein degradation pathways. In the autophagic process, vacuolar sorting protein 35 (VPS35) interacts and controls the trafficking of Wiskott–Aldrich syndrome protein (WASH) complex (blue) and autophagy-related protein 9A (ATG9A; blue) assuring the correct autophagosome formation; in chaperone-mediated autophagy (CMA), VPS35 controls the retrieval trafficking of lysosome-associated membrane protein 2A (LAMP2A) receptor (light green) to the trans-Golgi Network (TGN); VPS35 interacts with CI-MPR (dark green) and Sortilin (pink) leading indirectly to the trafficking of cathepsin D (CTSD, in red) through early endosomes (EE) and late endosomes (LE). VPS35–D620N mutation and VPS35 deficiency lead to dysfunctions in α-synuclein degradation. Figure was adapted from Servier Medical Art images (http://smart.servier.com/).

In normal conditions, the quality control network dealing with α-synuclein accumulation is efficient in promoting its refolding or degradation. In general, to induce α-synuclein degradation, a cell can activate the ubiquitin protein system or alternatively—in the presence of intracytoplasmatic aggregates and LBs—can enhance the endosome-lysosomal degradation system, macroautophagy, or CMA (52) (Figure 4). Recent studies highlighted that VPS35 can act at different levels of the α-synuclein degradation pathways (Figure 5). Particularly, VPS35 acts indirectly on lysosomal activity by sorting receptors of lysosomal hydrolases, including Sortilin and cation independent 6 mannose phosphate receptor (CI-MPR) (18). VPS35 regulates macroautophagy controlling the localization of WASH complex and the trafficking of ATG9a protein (15). Finally, VPS35 manages CMA by mediating the retrieval and transport of LAMP-2A receptor to lysosomes (16) (Figure 5).

### VPS35 Controls the Lysosomal Degradation Pathway

α-Synuclein enters through macroautophagy, CMA, and endosomal pathway into lysosome to be degraded by lysosomal hydrolases (53). VPS35 carries several cargoes involved in neurodegenerative disorders and more specifically in the clearance of α-synuclein (48). Two well-studied VPS35 cargoes are Sortilin (20, 21) and CI-MPR (18, 19), and VPS35–D620N mutant showed defects in sorting CI-MPR (54). These two cargoes operate as lysosomal hydrolase receptors, sorting acid hydrolases, such as cathepsins, from TGN to lysosomes. Aspartyl protease (cathepsin D) is the main lysosomal endopeptidase responsible for the degradation of α-synuclein (55). However, cysteine proteases (cathepsin B and L) have been shown to be effective in degraded α-synuclein (53). Direct interaction between α-synuclein and lysosomal proteases was shown in mice and human purified lysosomal extracts (53). CTSD-deficient mice showed accumulation of insoluble α-synuclein aggregates in the brain (55). Accordingly, CTSD-mutant brain from mice, sheep, and human showed selective synucleinopathy-like changes as well as α-synuclein accumulation and formation of ubiquitin-positive inclusions (55). Moreover, haploinsufficiency of CTSD resulted in the reduction in lysosomal functions and also acceleration of the propagation of LB pathology (56). Furthermore, recent studies in *Drosophila* have shown a direct link between VPS35 depletion and defects in CTSD trafficking and maturation (17) (Figure 5).

### VPS35 Controls Macroautophagy

Macroautophagy has a profound importance in the clearance of intracytoplasmatic aggregate-prone proteins as well as α-synuclein in PD and huntingtin in Huntington's disease (57). The treatment with rapamycin, which stimulates autophagy, enhanced the clearance of aggregate-prone proteins (57). Dysregulation of the autophagy pathway has been observed in the brain of PD patients and in PD mouse models (58), and the overexpression of α-synuclein caused mislocalization of autophagic proteins and impaired macroautophagy (59).

VPS35 controls the endosomal recruitment of WASH complex, a scaffolding protein localized at the EE (15). WASH complex regulates (i) the autophagic process of the autophagosome development (15), (ii) the formation of actin patch on endosomes to promote protein sorting (60), and (iii) endosome-to-cell surface recycling (61). Recently, it has been demonstrated that retromer interacts directly with FAM21–WASH complex controlling its correct localization (15). VPS35–D620N mutant destabilized this interaction, impairing WASH complex recruitment to endosomes (15, 62). The mislocalization of WASH complex induces defects in autophagosome formation, perturbing protein sorting, and specifically altering ATG9A trafficking (15) (Figure 5). ATG9A is a multipass transmembrane protein that acts early in the autophagic pathway and controls LC3-positive compartment interaction with autophagosome (15). Moreover, parkin PD mutations could impair the ubiquitination of VPS35, inducing the regulation of retromer-dependent sorting (63). Thus, parkin deficiency leads to the reduction in retromer-associated WASH complex cargo in mouse brains (63).

### VPS35 Controls CMA

The clearance of misfolded proteins and aggregates by CMA is essential for the normal cellular functions and its efficiency decline with age. Defects in CMA have been detected in PD patient (64). Therefore, the protein levels of CMA markers (LAMP2A and hsc70) were significantly reduced in SNpc and amygdala of PD patients (64). CMA is controlling the degradation of cytosolic proteins (as well as α-synuclein and LRRK2) carrying the recognition motif (KFERQ) (41). CMA presents multiple steps: (i) the recognition of KFERQ motif by HSPA8/HSC70 which target the substrate to lysosomes, (ii) the binding of the substrate to LAMP2A receptor, (iii) formation of substrate–translocation complex with membrane-bound LAMP2A, (iv) substrate degradation by lysosomal enzymes, and (v) disassembly of translocation complex and degradation of multimeric LAMP2A to be recycled (41). Recent studies have been shown that LAMP2A receptor trafficking from endosome to Golgi is altered in mice with reduced VPS35 levels or VPS35–D620N mutations (Figure 5). These mice showed alterations in lysosomal morphology with an increase in LAMP1A, reduction in LAMP2A vesicles, and an a relative increase in α-synuclein accumulation (16) (Figure 5).

## Discussion And Outlook

Pathogenic mutations in different PD-related genes with autosomal dominant or autosomal recessive Mendelian inheritance (LRRK2, VPS35, DNAJC13, SYNJ1, TMEM230, RAB39B) are involved in endosomal trafficking, indicating that DA neurons are specifically susceptible to endosomal dysfunctions (36). Neuropathological and neuroanatomical studies have demonstrated that the impairment of cellular trafficking may preferentially affect SNc DA neurons (36, 65). VPS35 mutations identified in PD lead to retromer dysfunctions (29, 30). Recent studies highlighted the interplay between VPS35 and LRRK2, two endosomal proteins associate to late-onset PD, showing a common pathway for the sorting of proteins through TGN and endolysosomal system (66–68). The loss of VPS35 or LRRK2 in *Drosophila* has been reported to affect synaptic recycling (68). Furthermore, the overexpression of VPS35 or VPS26 retromer subunits ameliorated the pathogenic mutant LRRK2 eye phenotype, protecting from locomotor deficit (67). VPS35–D620N KI mice or knockdown of VPS35 mice impacted LRRK2-mediated Rab protein phosphorylation (44) supporting the hypothesis that VPS35–D620N mutation resulted in a gain of function (44).

Different studies have shown that VPS35 has a key role in controlling α-synuclein accumulation in different PD models (14, 16, 17) even if the development of VPS35–D620N KI mice lack the presence of α-synuclein accumulation (43). Here, we review evidence that the increase in VPS35 levels in neurons represents a potential therapeutic target for the treatment of PD. Boosting VPS35 functions could help DA neurons to degrade α-synuclein toxic aggregates and prevent neuronal loss (Figure 5).

**Figure 6:**
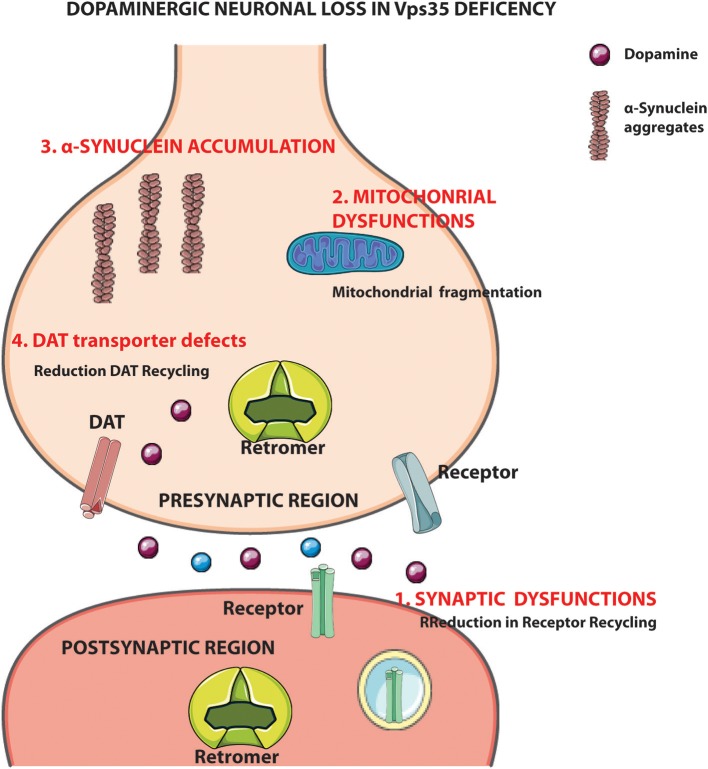
VPS35 deficiency and dopamine (DA) neuronal loss. VPS35-retromer subunit controls different mechanisms in the cells as well as synaptic and mitochondrial functions other than dopamine transporter (DAT) recycling and α-synuclein degradation. Altogether, the data give an overview about the possible molecular mechanisms controlled by vacuolar protein sorting 35 (VPS35), which could lead to DA neuron degeneration in Parkinson's disease (PD). Figure was adapted from Servier Medical Art images (http://smart.servier.com/).

Despite the understanding on VPS35 association to PD pathology, very little is known about the molecular mechanisms involved in the control of α-synuclein accumulation and in a possible neuroprotective role of VPS35 from α-synuclein-induced toxicity. The retromer complex is mainly involved in neurodegenerative disorders through the control of synaptic functions, trafficking, and localization of cargo proteins that are essential for neuronal homeostasis and α-synuclein accumulation vs. clearance. Therefore, VPS35 mutations could cause PD by acting on the α-synuclein degradation pathways (15–17, 69). A more detailed knowledge on the molecular mechanisms by which VPS35 promotes α-synuclein clearance could serve to clarify the role of VPS35-retromer protein in PD and to identify alternative and efficient new potential therapeutic targets downstream to VPS35 action. Besides, validation of new VPS35-based targets could be tested in PD mouse models before being developed in the clinical setting. VPS35 has a wide spectrum of actions within neurons, many of which are potentially relevant for the pathophysiology of PD, particularly synaptic functions (22–24, 26, 48) and control of DAT recycling (27) (Figure 6). Moreover, VPS35 controls mitochondrial function: VPS35 deficiency disrupts the mitophagic process, promoting mitochondrial fragmentation and DA neuron loss (28).

However, the role of VPS35 could also be played in a more general level on the control of accumulation/degradation of misfolded proteins, hence representing a potential target for different neurodegenerative disorders, such as PD and AD. Indeed, dysregulation of retromer transport is also strongly implicated in AD (70–73). Alterations of retromer activity in AD mouse models leads to pronounced neurodegeneration (deficit in post-synaptic glutamatergic neurotransmission) and deficiency in development of hippocampal-dependent memory (impairment of long-term potentiation) (36, 65) compatible with the role of retromer complex in controlling synaptic functions (22–24, 26). Moreover, different studies addressing VPS35 loss of function reported an elevation of endogenous Aβ peptide levels, in keeping with a VPS35 role in controlling amyloid protein precursor (APP) sorting and processing (28, 70–73).

New research may seek to develop a VPS35-based therapy. A possible therapeutic strategy to boost VPS35 functions could be a drug promoting the stabilization of retromer complex. Earlier studies identified chemical molecules capable of stabilizing the retromer complex by binding the complex in a bind site between VPS35–VPS29 (74). The stabilization of the retromer complex resulted in the reduction in complex degradation and in the increase in VPS35 and retromer subunit levels in cell lines (74). Starting from this observation, it could be possible to identify a lead compound able to combine the effect on the target with the possibility to cross the blood–brain barrier, to be tested in a clinical trial on PD patients. On the other hand, a second disease-modifying approach to boost VPS35 functions could be the delivery of VPS35 gene in the brain by adeno-associated-2-viral vectors (AAV2). The gene therapy approach will allow the introduction of VPS35 gene in the cells that result in the upregulation of VPS35 expression. Recently, it has been demonstrated that AAV2 treatment represents promising human therapeutic applications, with increasing evidence for safety and efficacy (75). First, AAV2 is the most effective in delivery genes *in vivo* for the presence of the primary adenovirus receptor in human cells that allow easy infection with a high yield of transgene expression. Moreover, currently, there are many clinical trials using AAV2 applications, and doses and routes of administration are already established. Finally, the elucidation of the molecular mechanism used by VPS35 to control neuronal functions, neuroprotection, and α-synuclein clearance could be addressed and may provide a new way to identify new therapeutic strategies in PD and other neurodegenerative disorders where neurotoxic aggregates build up. The other challenges will be to overcome in the translational therapeutic pipeline, including biomarker development, clinical trial strategies, and understanding the potential utility of VPS35 as therapeutic target in different PD backgrounds (sporadic and genetic PD cases).

A clinically related question is when a potentially disease-modifying therapy should be prescribed. Patients who are in the early symptomatic phase are thought to have a significant proportion of viable DA neurons. The diverse functions of VPS35 and its ability to counteract α-synuclein accumulation would justify testing VPS35-based approach from the prodromal until the early symptomatic phase of PD.

## Author Contributions

SE and AA conceived the study, wrote, and revised the manuscript. SE generated the figures.

### Conflict of Interest

The authors declare that the research was conducted in the absence of any commercial or financial relationships that could be construed as a potential conflict of interest.

## Abbreviations

AD: Alzheimer's disease
APP: amyloid protein precursor
ATG9A: autophagy-related protein 9A
CI-MPR: cation independent 6 mannose phosphate receptor
CMA: chaperone-mediated autophagy
CTSD: cathepsin D
DN: dopaminergic neurons
DAT: dopamine transporter
EE: Early endosome
LAMP2A: Lysosome-associated membrane protein 2A
PD: Parkinson's disease
SN: substantia nigra
TGN: trans-Golgi Network
WASH: Wiskott–Aldrich syndrome protein
VPS35: vacuolar sorting protein 35.
